# Awareness of disability among saudi university graduates

**DOI:** 10.1016/j.heliyon.2022.e11647

**Published:** 2022-11-16

**Authors:** Abdullah Madhesh

**Affiliations:** Special Education Department, Shaqra University, Shaqra, Saudi Arabia

**Keywords:** Disability, Disability awareness, People with disabilities, Saudi universities, University graduates

## Abstract

Awareness of disability is fundamental to creating equity for people with disabilities in any society. Because Saudi university graduates constitute an important part of society, this study aims to explore the level of awareness of disability amongst Saudi university graduates as well as the level of experience on disability as provided by their universities. A cross-sectional study was performed using a questionnaire developed by the researchers specifically for the purposes of this study. The questionnaire consisted of 4 sections, containing 39-items. Social media were used to recruit the participants and solicit responses: Twitter, Facebook and WhatsApp groups. Upon analysis of the results, it was observed among all respondents (n = 449, male = 246, female = 203) that there was a lack of disability awareness. As such, there is a need to increase this awareness among university students in Saudi Arabia. There is also a fundamental need to provide some programs and/or courses about disability in Saudi universities. Moreover, there were statistically significant differences between gender, university type and university major. The study was limited to the Saudi context, and perhaps further research can deal with the major reasons for the awareness shortcomings, and may also cover other countries, singularly or comparatively, which would in turn yield better and more effective results. The study helps address the equality of people with disabilities where the result of the study can be used as the basis for further research on the topic.

## 1 Introduction

Knowledge and awareness constitute essential components of any human being. More importantly, awareness is considered the main enhancer of human behavior. According to Schwartz (1968), awareness influences both human behavior and action. Wicklund (1979) also states that a person's awareness affects their actions to behave more consistently and to become more faithful to societal norms. Indeed, awareness affects human beliefs, attitudes and actions (Fabrigar et al., 2006). One of the most important types of awareness in human lives is awareness of disability. It is defined as educating the public about disability and people with disabilities from different angles such as their rights, appropriate treatment, and viewing them as equal citizens (Chae et al., 2019).

As a result, this study considers awareness of disability among people as a cornerstone for building more equitable societies. In addition, it concentrates on Saudi university graduates for two reasons. First, most high school students in Saudi Arabia complete their education and pursue bachelor's degrees. Second, those graduates are expected to occupy many important positions in the future, such as policymakers, teachers, and doctors, who will lead future actions or decisions that will touch the lives of people with disabilities either directly or indirectly.

### 1.1 Awareness of disability

There is no community devoid of people with disabilities. Thus, awareness of disability constitutes a cornerstone of any society that seeks to be developed and maintain the basic human rights of all its people. Indeed, some studies suggest that disability was constructed by society in many cases (Goode 2007; Heymani et al., 2020), e.g. the social model of disability. Awareness of disability is, simultaneously, essential for individuals and institutions. As such, disability awareness programs aim to raise disability knowledge that enhances positive attitudes and acceptance among the public towards disability and people with disabilities (McKay et al., 2015; McKay and Park 2019; Heymani et al., 2020). Awareness of disability is related to many fields and issues; however, this study will cover three main areas, namely awareness about the rights of people with disabilities, awareness about society's role towards people with disabilities, and awareness of disability at the university level.

### 1.2 Awareness about the rights of people with disabilities

People with disabilities face difficulties to achieve their basic human rights. They face exclusion, discrimination, oppression, inferior looks, neglect, negative attitudes and collective indifference (Slee 2011, 2018; Goodley and Runswick-Cole 2016; Barton 2018; Goodley 2020). Indeed, the rights of people with disabilities such as inclusion, empowerment, education, respect, acceptance, participation, and equality need to be met and upheld through a number of instruments, such as laws, policies, and societal norms. These instruments are based on spreading and publicizing the awareness of disability among all people regardless of their age, gender and education level. Generally, while these instruments may contribute to upholding the rights of people with disabilities, it is basically community's awareness of the existence of the needs and rights of this segment of society that enables them to feel as part and parcel of the community.

Awareness of disability plays a significant role in increasing the achievement of people with disabilities rights via changing inappropriate attitudes, behaviors and beliefs among people without disabilities. This was evidenced by a systematic review conducted by Armstrong et al. (2017) which covered 12 studies published between 2000 and 2012 about the impact of awareness of disability programs. The results showed improvements in students’ attitudes towards people with disabilities. Furthermore, a Korean study conducted by Lee and Han (2018) showed how a disability awareness program that was taught to 508 people, significantly affected their knowledge about discrimination and differentiation towards people with disabilities. Another study confirmed an immediate and positive influence of a short-term disability awareness program that was applied to 247 students on their knowledge and attitudes toward individuals with disabilities (Magnusson et al., 2017).

More recently, Chae et al. (2019) conducted a meta-analysis of 20 studies published between 2001 and 2017 on the effectiveness of intervention programs about disability awareness. They found a significant increase in acceptance and positive attitudes towards disability across all 20 studies.

### 1.3 Awareness about society's role towards people with disabilities

Society has an immense influence on the lives of people with disabilities in many ways. This is reflected in the social model of disability. This model stems from the principle that a person with disability is not disabled by his/her impairment but is disabled because of the barriers and obstacles s/he faces in society (Oliver 2013). Indeed, the Disability Studies in Education (DSE) rely on the concept of this model instead of concentrating on the medical model of disability that sees the disability as a medical problem that needs to be fixed (Collins and Ferri 2016).

Society means a lot to people with disabilities because they spend most of their time interacting with various institutions, individuals and elements. Unfortunately, this group of people faces many obstacles, challenges and difficulties in society. Barton (2018) states that in modern societies a person with disability is more vulnerable to discrimination and exclusion. Despite international legislation and laws that call for preserving the rights of individuals with disabilities to inclusion, education and equal life, exclusionary practices are still prevalent in many social systems around the globe (Whitburn 2015). In addition, Slee (2011) confirms that the collective indifference towards disability refers to the ways that people in societies have adopted to ignore the suffering that is spreading around us. Confronting and reducing this collective indifference and such inappropriate practices begins by spreading awareness among communities about their roles towards people with disabilities.

Society's role contributes to fostering societal norms and programs that enhance equal treatment of individuals with disability. This awareness shines a light on negative social attitudes and environmental impediments that stand as pernicious barriers that face individuals with disabilities (Dirth and Branscombe 2017). Increasing awareness among the members of society about their role in facilitating many aspects in the lives of people with disabilities enhances positive community participation and reduces harmful behaviors targeting or affecting persons with disabilities. One of the most important means of spreading this awareness is education, and one of its most important stages is university education.

### 1.4 Experiences with disability at the university level

University education is an important stage in individuals’ lives, particularly those who choose to pursue their education after school. "The university is the place where everything we think we know can be challenged and where new ideas are generated and transmitted" (Willetts, 2017: p1). The graduates of universities usually become the leaders, policymakers, teachers and doctors of the future. In this capacity, their futures affect people with disabilities either directly and/or indirectly. Thus, the university curricula must have adequate disability awareness programs. Indeed, implementing disability awareness programs at the university level enhances the acceptance and inclusion of persons with disabilities, increase positive attitudes towards students with disability, and decreases discomfort when interacting with people with disabilities (Santiago et al., 2016; McKay and Park 2019; Kirk et al., 2021). This was evidenced in a study conducted by Park and Kim (2018), who examined the effectiveness of a disability awareness program on 30 university students and found significant changes in their attitudes towards people with disabilities upon completion of the program.

Another study conducted by Yotsumoto et al. (2010) developed an awareness program that was implemented by individuals with disabilities. University students, professionals and community members attended this program and the findings of this study showed that there were positive and significant changes in attitudes, acceptance and feelings of all the participants. A more recent study conducted by Iwakuma et al. (2021) examined the impact of a university program called “What is Disability” on freshman university students. This program aimed to increase the awareness of disability among students and the results of this study concluded that there was a decrease in stereotypes about disability among participants and their attitudes towards people with disabilities became more positive.

However, many universities do not give these programs enough space in their curricula. Heymani et al. (2020) state that some universities may advocate the rights of people with disabilities in education and inclusion, but disability awareness programs may not be their priority and primary focus. Higher education institutions need to increase the content of disability awareness when designing their curricula to achieve inclusivity for people with disabilities (Fabrigar et al., 2006).

Despite that many studies have examined the impact of different disability awareness programs at universities on disability awareness, the researchers did not find any study that investigates disability awareness among university graduates, particularly in the Saudi environment. As a result, this study aims to explore the level of awareness of disability amongst Saudi university graduates as well as the level of their experience on disability as provided by their universities.

### 1.5 Research questions

This study includes the five following questions:

1. What is the level of disability awareness among Saudi university graduates?
2. What is the level of awareness of the rights of people with disabilities among Saudi university graduates?
3. What is the level of awareness about society's role towards people with disabilities among graduates of Saudi universities?
4. What is the level of experience on disability provided to Saudi university graduates?
5. Are there statistically significant differences between the mean averages of the study sample responses on each dimension of the study tool, and on the study tool as a whole, based on the variables of university type, gender, and university major?

## 2 Material and methods

This section addresses the materials used in the study, the method used for data collection, and the test used in the research study.

### 2.1 Study design

#### 2.1.1 Participants

A cross-sectional study was conducted from October to December 2021. Participation was totally voluntary and unpaid. and the participants had to provide their informed consent before starting their responses. Random sampling method was used to invite the participants to contribute to the study electronically. The following inclusion criteria were applied: (1) The participant graduated from a Saudi public university (42 universities), (2) The participant graduated last year (i.e. 2021). Based on these criteria, the population of this study is roughly 29,000 graduates (Saudi Open Data, 2021).

To select a representative sample of the population, the Stephen Thompson equation was applied, and a total of 600 participants responded. A number of 151 (%25.2) responses was excluded from the study because they did not meet the inclusion criteria of this study. A total of 449 (%74.8) questionnaires were complete and fit for analysis (n = 449, male = 246, female = 203). The following subsection describes the demographic information of participants:

#### 2.1.2 University type

It is clear from Table (1) that 431 of the study respondents, who represent 96.0% of the study sample, are graduates of public universities, whereas 18 of the study respondents, who represent 4.0% of the study sample, graduated from private universities.

#### 2.1.3 Gender

As seen in Table (1), 246 of the study respondents, standing for 54.8% of the study sample, are males whereas 203 respondents (i.e. 45.2% of the study sample) are females.

#### 2.1.4 University major

It is clear from Table (1) that 226 of the respondents (50.3% of the study sample) studied humanities while 223 of the respondents (49.7% of the study sample) had scientific majors.

Grade Point Average (GPA) As can be seen from Table (1), 165 of the study respondents (i.e. 36.7% of the study sample) had very good GPA; 151 respondents (33.6% of the study sample) had good GPA; while 105 respondents (representing 23.4% of the study sample) had excellent GPA. Additionally, 28 of the study respondents (6.2% of the study sample) passed with average grade (Table 1).

#### 2.1.5 Ethical approval

For the purposes of this study, ethical approval was sought and received from Shaqra University Research Ethics Committee (No: ERC_SU_20210053), attached in the appendix.

#### 2.1.6 Instruments/measurement

The principal measurement tool used in this study was a questionnaire. The questionnaire was created by the researchers in the form of paper-based copies based on literature reviews, such as (Heymani et al., 2020; Roth et al., 2018; Series, 2019; Wynants and Dennis, 2017). For validation, these copies were sent to six colleagues and academics specialized in education. This stage resulted in some minor changes which helped to improve the questionnaire. To ensure that the instrument did not contain incomprehensible or vague questions, it was pilot-tested with 15 university graduates and modified based on their comments.

The variables/items were tested for their reliability and validity, and the measurement coefficients were supportive. The final questionnaire was written electronically in Google Forms and was socialized by WhatsApp and sent to 600 Saudi university graduates. This questionnaire had four subscales: “the level of disability awareness among Saudi university graduates,” which consisted of 12 statements/variables (from 1 to 12); “level of awareness of the rights of people with disabilities among graduates of Saudi universities,” which consisted of nine statements/variables (from 13 to 21); “the level of awareness about society's role towards people with disabilities” which consisted of nine statements (from 22 to 30); and “university experiences regarding disability provided to graduates of Saudi universities,” which consisted of nine statements (from 31 to 39).

After reading the study's information and clicking the participation option, the respondents provided primary demographic data (university type, gender, university major and Grade Point Average (GPA)). Out of the 600 questionnaires, a total of 449 responses were complete and eligible for analysis. Responses were scored using a five-point Likert scale ranging from strongly disagree (1), disagree (2), neutral (3), agree (4) and strongly agree (5).

### 2.2 Data collection procedure

As part of Shaqra University research policies, researchers must adhere strictly to a set of ethical rules and must demonstrate that prior to obtaining approval for publications. All participants were informed of their roles as voluntary informants and that the data they provided was confidential and strictly used for research purposes. Secondly, to garner the power of technology, social media (websites and applications) were invested in the distribution and collection of questionnaires, thus providing ease for respondents as well as researchers, and ensuring meticulous data collection procedures.

### 2.3 Data analysis

To achieve the objectives of this study and analysis of the collected data, the researchers used the Statistical Package for Social Sciences (SPSS). After the data was encoded and entered into the computer, the researchers determined the length of cells of the 5th scale. The researchers calculated the range (5–1 = 4) and then divided it by the number of the scale's cells to get the correct cell length (4/5 = 0.80). After that, the researchers added this value to the lowest value in the scale (or the beginning of the scale which is the correct one) in order to determine the upper limit of this cell, and thus the length of the cells became as follows:

(1) From 1 to 1.80 represents (Strongly disagree)
(2) From 1.81 to 2.60 represents (Disagree)
(3) From 2.61 to 3.40 represents (Neutral)
(4) From 3.41 to 4.20 represents (Agree)
(5) From 4.21 to 5 represents (Strongly agree).

The frequencies and percentages were calculated in order to identify the variables of this study based on the personal and academic characteristics of the study sample (university type, gender, university major and Grade Point Average (GPA)) and to identify the responses to the main items as well.

Meanwhile, the statistical measures calculated include the Weighted Mean, the Mean, the Standard Deviation, t-test and One-Way ANNOVA.

## 3 Results

### 3.1 Research questions results

#### 3.1.1 The results of the first question

To answer, “What is the level of disability awareness among Saudi university graduates?“, the frequencies, percentages, means, standard deviations, and ranks of the responses of study respondents were calculated for each item as can be seen in Table 2.

**Table 2:** Level of disability awareness among Saudi university graduates.

N	Phrase	Strongly Disagree		Disagree		Neutral		Agree		Strongly agree		Mean	Std. Deviation	Rank	response
		F	%	F	%	F	%	F	%	F	%				
1	I can distinguish the various types of disability.	13	2.9	30	6.7	81	18	215	47.9	110	24.5	3.84	0.96	6	Agree
2	I am aware of disability groups.	14	3.1	62	13.8	83	18.5	204	45.4	86	19.2	3.64	1.04	8	Agree
3	I think that all people with disabilities constitute one group.	152	33.9	186	41.4	43	9.6	51	11.4	17	3.8	2.10	1.11	12	Disagree
4	In my opinion, people with disabilities face difficulty interacting with people.	12	2.7	54	12	102	22.7	180	40.1	101	22.5	3.68	1.04	7	Agree
5	I believe that people with disabilities can learn.	2	0.4	11	2.4	16	3.6	145	32.3	275	61.2	4.51	0.72	2	Strongly agree
6	I think that a person with a disability can pursue a certain profession.	1	0.2	24	5.3	44	9.8	178	39.6	202	45	4.24	0.85	4	Strongly agree
7	I believe that marriage between relatives is one of the causes of disability.	24	5.3	54	12	135	30.1	140	31.2	96	21.4	3.51	1.11	9	Agree
8	I believe that people with disabilities can benefit from modern technology.	1	0.2	5	1.1	23	5.1	137	30.5	283	63	4.55	0.67	1	Strongly agree
9	All people with disabilities depend on the help of others.	52	11.6	176	39.2	118	26.3	74	16.5	29	6.5	2.67	1.08	11	Neutral
10	I believe that there are people with disabilities who have special talents.	5	1.1	19	4.2	24	5.3	143	31.8	258	57.5	4.40	0.86	3	Strongly agree
11	I know how to deal with people with disabilities.	41	9.1	87	19.4	125	27.8	152	33.9	44	9.8	3.16	1.13	10	Neutral
12	I feel that I need to increase my knowledge of disability categories.	5	1.1	11	2.4	50	11.1	197	43.9	186	41.4	4.22	0.82	5	Strongly agree
Mean												3.71	0.37	Agree

**Table 1:** The demographic information of participants.

University type	Frequency	Percent
Public	431	96.0 %
Private	18	4.0%
Total	449	100 %
**Gender**	**Frequency**	**Percent**
Male	246	54.8%
Female	203	45.2%
Total	449	100 %
**University Major**	**Frequency**	**Percent**
Scientific	223	49.7%
Theoretical	226	50.3%
Total	449	100 %
**Grade Point Average (GPA)**	**Frequency**	**Percent**
Excellent (A)	105	23.4%
Very good (B)	165	36.7%
Good (C)	151	33.6%
Acceptable (D)	28	6.2%
Total	449	100 %

The results of the level of disability awareness among Saudi university graduates are displayed in Table 2. Clearly, the table shows that there is a convergence in the responses (the level of disability awareness among Saudi university graduates). Additionally, the general mean on the statements of the axis is (3.71), and the mean is put at the fourth class of the 5^th^ scale, whose average ranges from (3.41–4.20) refers to the “agree” measurement. Also, it was noticed that the statements concerning the level of disability awareness among Saudi university graduates can be arranged in descending order:

(6) Statement (8), i.e. “I believe that people with disabilities can benefit from modern technology”, appears in 1^st^ position with most respondents choosing “strongly agree” (M = 4.55, SD = 0.67).
(7) Statement (5), i.e. “I believe that people with disabilities can learn”, comes in the 2^nd^ position with most respondents choosing “strongly agree” (M = 4.51, SD = 0.72).
(8) Statement (10), i.e. “I believe that there are people with disabilities who have special talents”, comes in the 3^rd^ position with most respondents choosing “strongly agree” (M = 4.40, SD = 0.86).
(9) Statement (6), i.e. “I think that a person with a disability can pursue a certain profession”, occurs the 4^th^ position with most respondents choosing “strongly agree” (M = 4.24, SD = 0.85).
(10) Statement (12), i.e. “I feel that I need to increase my knowledge of disability categories”, has occupied the 5^th^ position with most respondents choosing “strongly agree” (M = 4.24, SD = 0.82).
(11) Statement (1), i.e. “I can distinguish the various types of disability”, took the 6^th^ position with most respondents choosing “agree” (M = 3.84, SD = 0.96).
(12) Statement (4), i.e. “In my opinion, people with disabilities face difficulty interacting with people”, comes in the 7^th^ position with most respondents choosing “agree” (M = 3.68, SD = 1.04).
(13) Statement (2), i.e. “I am aware of disability groups”, occurs in the 8^th^ position with most respondents choosing “agree” (M = 3.64, SD = 1.04).
(14) Statement (7), i.e. “I believe that marriage between relatives is one of the causes of disability”, appears in the 9^th^ position with most respondents choosing “agree” (M = 3.51, SD = 1.11).
(15) Statement (11), i.e. “I know how to deal with people with disabilities”, happens in the 10^th^ position with most respondents choosing “agree” (M = 3.16, SD = 1.13).
(16) Statement (9), i.e. “All people with disabilities depend on the help of others”, appears in the 11^th^ position with most respondents choosing “neutral” (M = 2.67, SD = 1.08).
(17) Statement (3), i.e. “I think that all people with disabilities constitute one group”, occurs in the 12^th^ position with most respondents choosing “disagree” (M = 2.10, SD = 1.11).

I can conclude that the general mean of the level of disability awareness among Saudi university graduates is 3.71 out of 5. This means that most Saudi university graduates have an average level of awareness about disability as most believe that:

• Persons with disabilities can benefit from modern technology.
• Persons with disabilities can learn.
• There are persons with disabilities who have special talents.
• A person with disability can pursue a certain profession.
• There is a need to increase the knowledge about disability categories.
• The respondents can distinguish the various types of disability.
• People with disabilities face difficulty interacting with other people.

#### 3.1.2 The results of the second question

To answer the questions of “What is the level of awareness of the rights of people with disabilities among Saudi university graduates?“, the frequencies, percentages, means, standard deviations, and ranks of the responses were calculated, and are presented in the table below.

The results of the level of awareness of the rights of people with disabilities among graduates of Saudi universities are displayed in Table 3. Clearly, the table shows that there is a convergence of responses on the awareness of the rights of people with disabilities among graduates of Saudi universities. Additionally, the general mean on the statements of the axis is 3.22, which puts it at the third position of the 5^th^ scale, whose average ranges from 2.61 to 3.40 – i.e. this highlights the “neutral” position. The statements concerning the awareness of the rights of people with disabilities among graduates of Saudi universities are arranged below in a descending order:

(18) Statement (18), i.e. “If I were a recruitment officer, I would hire people with disabilities for appropriate positions”, appears in the 1st position with most respondents choosing “agree” (M = 4.17, SD = 0.76).
(19) Statement (14), i.e. “I think that the Ministry of Social Affairs should take care of the rights of people with disabilities”, occupies the 2nd position with most respondents choosing “agree” (M = 4.03, SD = 0.94).
(20) Statement (16), i.e. “I am not familiar with the international conventions on disability”, happens in the 3rd position with most respondents choosing “agree” (M = 3.93, SD = 1.40).
(21) Statement (20), i.e. “To protect the rights of students with disabilities, they should be placed in special schools or classes”, takes the 4^th^ position with most respondents choosing “neutral” (M = 3.27, SD = 1.27).
(22) Statement (17), i.e. “I feel pity for people with disabilities when I see their behavior”, occurs in the 5th position with most respondents choosing “neutral” (M = 3.25, SD = 1.23).
(23) Statement (19), i.e. “I believe it is more relevant to look after the rights of ordinary people because they are the majority in society”, comes in the 6th position with most respondents choosing “neutral” (M = 2.93, SD = 1.27).
(24) Statement (15), i.e. “I do not know whether or not there is a system for the care of people with disabilities in KSA”, happens to be in the 7th position with most respondents choosing “neutral” (M = 2.87, SD = 1.26).
(25) Statement (13), i.e. “I think that families should bear the sole responsibility for caring for their members with disabilities”, takes at the 8th position with most respondents choosing “disagree” (M = 2.56, SD = 1.18).
(26) Statement (21), i.e. “I don't mind giving a lower salary to an employee with disability because his abilities are less than the average employee”, appears in the 9th position with most respondents choosing “disagree” (M = 2.00, SD = 1.10).

**Table 3:** The level of awareness on the rights of people with disabilities.

N	Phrase	Strongly Disagree		Disagree		Neutral		Agree		Strongly agree		Mean	Std. Deviation	Rank	response
		F	%	F	%	F	%	F	%	F	%				
13	I think that families should bear the sole responsibility for caring for their members with disabilities.	80	17.8	183	40.8	73	16.3	80	17.8	33	7.3	2.56	1.18	8	Disagree
14	I think that the Ministry of Social Affairs should take care of the rights of people with disabilities.	7	1.6	30	6.7	61	13.6	196	43.7	155	34.5	4.03	0.94	2	Agree
15	I do not know whether or not there is a system for the care of people with disabilities in KSA.	57	12.7	156	34.7	86	19.2	88	19.6	62	13.8	2.87	1.26	7	Neutral
16	I am not familiar with the international conventions on disability.	12	2.7	37	8.2	76	16.9	169	37.6	155	34.5	3.93	1.04	3	Agree
17	I feel pity for people with disabilities when I see their behavior.	48	10.7	90	20	74	16.5	174	38.8	63	14	3.25	1.23	5	Neutral
18	If I were a recruitment officer, I would hire persons with disabilities for appropriate positions.	2	0.4	11	2.4	53	11.8	227	50.6	156	34.7	4.17	0.76	1	Agree
19	I believe it is more relevant to look after the rights of ordinary people because they are the majority in society.	64	14.3	123	27.4	105	23.4	93	20.7	64	14.3	2.93	1.27	6	Neutral
20	To protect the rights of students with disabilities, they should be placed in special schools or classes.	48	10.7	82	18.3	111	24.7	115	25.6	93	20.7	3.27	1.27	4	Neutral
21	I don't mind giving a lower salary to an employee with a disability because his abilities are less than the average employee.	188	41.9	144	32.1	59	13.1	46	10.2	12	2.7	2.00	1.10	9	Disagree
Mean												3.22	0.55	Neutral	

I can conclude that the general mean for the level of awareness of the rights of people with disabilities among the graduates of Saudi universities is 3.22 out of 5. This comes in the third position of the 5^th^ scale. It means that Saudi university graduates are neutral regarding the level of awareness of the rights of people with disabilities.

#### 3.1.3 The results of the third question

To answer the question of “What is the level of awareness about society's role towards people with disabilities among graduates of Saudi universities?”, the frequencies, percentages, means, standard deviations, and ranks of the responses were calculated, and are presented in the following table.

The results of the level of awareness about society role towards people with disabilities among the graduates of Saudi universities are displayed in Table 4. Clearly, the table shows that there is a convergence of responses regarding the level of awareness about society's role towards people with disabilities among the graduates of Saudi universities). Additionally, the general mean on the statements of the axis is 3.32, putting it in the third position of the 5^th^ scale, whose average ranges from 2.61 to 3.40 and refers to “neutral”. The statements concerning the level of awareness about society's role towards people with disabilities among the graduates of Saudi universities are arranged in a descending order:

(27) Statement (27), i.e. “It is important for everyone in society to realize their responsibility towards people with disabilities”, appears in the 1^st^ position with most respondents choosing “strongly agree” (M = 4.49, SD = 0.71).
(28) Statement (29), i.e. “I believe it is important to continue medical diagnosis and treatment for people with disabilities”, takes the 2^nd^ position with most respondents choosing “strongly agree” (M = 4.48, SD = 0.67).
(29) Statement (23), i.e. “Society's care for people with disabilities should be greater than for ordinary people”, happens in the 3^rd^ position with most respondents choosing “agree” (M = 4.04, SD = 0.92).
(30) Statement (26), i.e. “Society's responsibility towards people with disabilities begins after the disability occurs, not before that”, occupies the 4^th^ position with most respondents choosing “neutral” (M = 3.22, SD = 1.20).
(31) Statement (24), i.e. “The type, severity, and time of occurrence of disability should not be taken into consideration as determinants of the degree of community's care for people with disabilities”, occurs in the 5^th^ position with most respondents choosing “neutral” (M = 3.18, SD = 1.28).
(32) Statement (25), i.e. “The rights of people with disabilities can be separated from the rights of ordinary members of society”, occupies the 6^th^ position with most respondents choosing “neutral” (M = 3.10, SD = 1.23).
(33) Statement (30), i.e. “I am well aware of institutions and agencies that provide various services to people with disabilities”, takes the 7^th^ position with most respondents choosing “neutral” (M = 2.66, SD = 1.18).
(34) Statement (28), i.e. “Fulfilling society's responsibility towards people with disabilities is the task of specialists and stakeholders only”, appears in the 8^th^ position with most respondents choosing “disagree” (M = 2.45, SD = 1.24).
(35) Statement (22), i.e. “I think that society's responsibility towards people with disabilities should be limited to providing shelter and food”, occurs in the 9^th^ position with most respondents choosing “disagree” (M = 2.23, SD = 1.20).

**Table 4:** The level of awareness about society's role towards people with disabilities.

N	Phrase	Strongly Disagree		Disagree		Neutral		Agree		Strongly agree		Mean	Std. Deviation	Rank	response
		F	%	F	%	F	%	F	%	F	%				
22	I think that society's responsibility towards people with disabilities should be limited to providing shelter and food.	143	31.8	171	38.1	50	11.1	57	12.7	28	6.2	2.23	1.20	9	Disagree
23	Society's care for people with disabilities should be greater than for ordinary people.	7	1.6	17	3.8	90	20	173	38.5	162	36.1	4.04	0.92	3	agree
24	The type, severity, and time of occurrence of disability should not be taken into consideration as determinants of the degree of community's care for people with disabilities.	44	9.8	120	26.7	80	17.8	122	27.2	83	18.5	3.18	1.28	5	Neutral
25	The rights of people with disabilities can be separated from the rights of ordinary members of society.	50	11.1	100	22.3	120	26.7	113	25.2	66	14.7	3.10	1.23	6	Neutral
26	Society's responsibility towards people with disabilities begins after the disability occurs, not before that.	40	8.9	98	21.8	102	22.7	143	31.8	66	14.7	3.22	1.20	4	Neutral
27	It is important for everyone in society to realize their responsibility towards people with disabilities.	5	1.1	2	0.4	21	4.7	163	36.3	258	57.5	4.49	0.71	1	Strongly agree
28	Fulfilling society's responsibility towards people with disabilities is the task of specialists and stakeholders only.	100	22.3	188	41.9	66	14.7	49	10.9	46	10.2	2.45	1.24	8	Disagree
29	I believe it is important to continue medical diagnosis and treatment for people with disabilities.	2	0.4	1	0.2	29	6.5	165	36.7	252	56.1	4.48	0.67	2	Strongly agree
30	I am well aware of institutions and agencies that provide various services to people with disabilities.	74	16.5	155	34.5	107	23.8	76	16.9	37	8.2	2.66	1.18	7	Neutral
Mean												3.32	0.54	Neutral	

I can conclude that the general mean of the level of awareness about society's role towards people with disabilities among Saudi university graduates is 3.32 out of 5, putting it at the third position of the 5^th^ scale. This means that Saudi university graduates are neutral on the level of awareness about society's role towards people with disabilities.

#### 3.1.4 The results of the fourth question

To determine the level of experience on disability provided to Saudi university graduates, the frequencies, percentages, means, standard deviations, and ranks of the responses were calculated and are presented in the following table.

The results for the level of experience on disability provided to graduates of Saudi universities are displayed in Table 5. Clearly, the table shows that there is a convergence of responses on university experiences on disability provided by Saudi universities. Additionally, the general mean on the statements of the axis is 3.17, and this puts it at the third position of the 5^th^ scale, whose average ranges from 2.61 to 3.40, which refers to “neutral”. The statements concerning the level of experience on disability provided by Saudi universities are arranged in a descending order:

(36) Statement (39), i.e. “I was hoping to have community volunteer programs conducted by my university for people with disabilities and their families”, comes in the 1st position with most respondents choosing “strongly agree” (M = 4.24, SD = 0.89).
(37) Statement (38), i.e. “It is necessary to have university courses that develop students' awareness of disability and the rights of people with disabilities”, takes the 2nd position with most respondents choosing “agree” (M = 4.12, SD = 0.96).
(38) Statement (34), i.e. “I wish that some of the graduation projects in my academic major were related to serving people with disabilities”, happens in 3rd position with most respondents choosing “agree” (M = 3.42, SD = 0.95).
(39) Statement (35), i.e. “I observed that among the advertisements for events and activities in the college where I studied, there were items related to disability”, appears in the 4th position with most respondents choosing “neutral” (M = 2.89, SD = 1.28).
(40) Statement (33), i.e. “There is one or more people with disabilities among my colleagues”, occurs in the 5th position with most respondents choosing “neutral” (M = 2.86, SD = 1.36).
(41) Statement (32), i.e. “During my university study, I attended events in which people with disabilities participated”, appears in the 6th position with most respondents choosing “neutral” (M = 2.79, SD = 1.40).
(42) Statement (36), i.e. “The university where I studied offers many courses and programs related to people with disabilities”, occupies the 7th position with most respondents choosing “neutral” (M = 2.72, SD = 1.27).
(43) Statement (31), i.e. “The study plan in my university include disability courses”, takes the 8th position with most respondents choosing “disagree” (M = 2.55, SD = 1.34).
(44) Statement (37), i.e. “I believe that adding university courses on disability increases students' burden”, happens in the 9th position with most respondents choosing “disagree” (M = 2.51, SD = 1.19).

**Table 5:** Level of experience on disability provided by Saudi universities.

N	Phrase	Strongly Disagree		Disagree		Neutral		Agree		Strongly agree		Mean	Std. Deviation	Rank	response
		F	%	F	%	F	%	F	%	F	%				
31	The study plan in my university includes disability courses.	125	27.8	125	27.8	69	15.4	85	18.9	45	10	2.55	1.34	8	Disagree
32	During my university study, I attended events in which people with disabilities participated.	105	23.4	113	25.2	66	14.7	100	22.3	65	14.5	2.79	1.40	6	Neutral
33	There is one or more people with disabilities among my colleagues.	86	19.2	128	28.5	59	13.1	114	25.4	62	13.8	2.86	1.36	5	Neutral
34	I wish that some of the graduation projects in my academic major were related to serving people with disabilities.	9	2	31	6.9	105	23.4	193	43	111	24.7	3.82	0.95	3	agree
35	I observed that among the advertisements for events and activities in the college where I studied, there were items related to disability.	74	16.5	117	26.1	102	22.7	98	21.8	58	12.9	2.89	1.28	4	Neutral
36	The university where I studied offers many courses and programs related to people with disabilities.	89	19.8	128	28.5	100	22.3	83	18.5	49	10.9	2.72	1.27	7	Neutral
37	I believe that adding university courses on disability increases students' burden.	91	20.3	172	38.3	89	19.8	61	13.6	36	8	2.51	1.19	9	Disagree
38	It is necessary to have university courses that develop students' awareness of disability and the rights of people with disabilities.	10	2.2	21	4.7	62	13.8	170	37.9	186	41.4	4.12	0.96	2	agree
39	I was hoping to have community volunteer programs conducted by my university for people with disabilities and their families.	7	1.6	11	2.4	62	13.8	154	34.3	215	47.9	4.24	0.89	1	Strongly agree
Mean												3.17	0.67	Neutral	

We can conclude that the general mean of the level of experiences on disability provided by Saudi universities is 3.17 out of 5, putting it in the third position of the 5^th^ scale, which refers to “neutral”. This means that Saudi university graduates are neutral on the extent of university experiences on disability provided by Saudi universities.

#### 3.1.5 The result of the fifth question

To determine if there were statistically significant differences between the mean averages of study sample responses on each dimension of the study tool, and on the study tool as a whole, based on the variables of university type, gender, and university major, the researchers used an independent sample t-test. The results are presented in Table 6.

**Table 6:** University type, Gender, Grade Point Average (GPA).

University type							
Axis	Group	N	Mean	Std. Deviation	T	df	Sig
The level of disability awareness among Saudi university graduates	Public	431	3.72	0.37	3.28	447	0.01
	Private	18	3.43	0.43			
Level of awareness of the rights of people with disabilities among graduates of Saudi universities	Public	431	3.21	0.55	2.85	447	0.01
	Private	18	3.59	0.65			
The level of awareness about society's role towards people with disabilities among graduates of Saudi universities	Public	431	3.31	0.54	1.19	447	0.23
	Private	18	3.46	0.54			
Level of experience on disability provided to graduates of Saudi universities	Public	431	3.18	0.67	2.22	447	0.01
	Private	18	2.83	0.49			
**Gender**							
Axis	Gender	N	Mean	Std. Deviation	T	df	Sig
The level of disability awareness among Saudi university graduates	Male	246	3.74	0.36	1.92	447	0.06
	Female	203	3.67	0.39			
Level of awareness of the rights of people with disabilities among graduates of Saudi universities	Male	246	3.25	0.58	1.10	447	0.27
	Female	203	3.19	0.53			
Level of awareness about society role towards people with disabilities among graduates of Saudi universities	Male	246	3.33	0.58	0.76	447	0.45
	Female	203	3.29	0.48			
Level of experience on disability provided by Saudi universities	Male	246	3.23	0.65	2.35	447	0.02
	Female	203	3.09	0.67			
**Grade Point Average (GPA)**							
			Sum of Squares	Df	Mean Square	F	Sig.
The level of disability awareness among Saudi university graduates	Between Groups		1.29	3.00	0.43	2.11	0.07
	Within Groups		61.32	445.00	0.14		
	Total		62.61	448.00			
Level of awareness of the rights of people with disabilities among graduates of Saudi universities	Between Groups		0.67	3.00	0.22	0.72	0.54
	Within Groups		137.21	445.00	0.31		
	Total		137.87	448.00			
The level of awareness about society role towards people with disabilities among graduates of Saudi universities	Between Groups		1.34	3.00	0.45	1.56	0.20
	Within Groups		127.23	445.00	0.29		
	Total		128.57	448.00			
Level of experience on disability provided by Saudi universities	Between Groups		0.61	3.00	0.20	0.46	0.71
	Within Groups		197.98	445.00	0.44		
	Total		198.60	448.00			

Table 6 clearly shows a statistically significant difference in the level of awareness on disability between graduates from private universities (M = 3.43, SD = 0.43) and public universities (M = 3.72. SD = 0.)37, with those from public universities having greater awareness levels, i.e. *t* (447) = 3.28. *p* = 0.01.

There was also a statistical difference in the level of awareness on the rights of people with disabilities between graduates from private universities (M = 3.59, SD = 0.65) and public universities (M = 3.21. SD = 0.55), with those from private universities having greater awareness levels, i.e. *t* (447) = 2.85. *p* = 0. 01.

There was no statistical difference between private university graduates (M = 3.46, SD = 0.54) and public university graduates (M = 3.31, SD = 0.55) regarding the level of awareness about society's role towards people with disabilities – *t* (447) = 1.19, *p* = 0.23).

There was a statistical difference between public university graduates (M 3.18, SD = 0.67) and private university graduates (M = 2.83, SD = 0.49) regarding the level of experience on disability provided by the universities: *t* (447) = 2.22, *p* = 0.01). As can be seen, public universities offered more experiences.

As can be seen from Table 6, there was no statistical difference between males (M = 3.74, SD = 0.36) and females (M = 3.67, SD = 0.39) regarding the level of awareness on disability *t(*447) = 1.92, *p* = 0.06. There was also no statistical difference between males (M = 3.25, SD = 0.58) and females (M = 3.19, SD = 0.53) regarding the level of awareness of the rights of people with disabilities *t(*447) = 1.10, *p* = 0.27. There was no statistical difference between males (M = 3.33, SD = 0.58) and females (M = 3.29, SD = 0.48) regarding the level of awareness about society's role towards people with disabilities *t(*447) = 0.76, *p* = 0.45. Finally, there was a statistical difference between males (M = 3.23, SD = 0.65) and females (M = 3.09, SD = 0.67) regarding the level of experience on disability provided by the universities *t(*447) = 2.35, *p* = 0.02. As can be seen males had more experiences with disability than females.

As can be seen from Table 6, there was no statistical difference between the science majors (M = 3.70, SD = 0.40) and those with a theoretical major (M = 3.72, SD = 0.34) regarding the level of awareness on disability *t(*447) = 0.33, *p* = 0. 74.

There was no statistical difference between the science majors (M = 3.25, SD = 0.57) and those with a theoretical major (M = 3.20, SD = 0.54) regarding the level of awareness of the rights of people with disabilities *t(*447) = 1.06, *p* = 0.29.

There was also no statistical difference between the science majors (M = 3.32, SD = 0.54) and those who studied humanities (M = 3.31, SD = 0.54) regarding the level of awareness about the role of society towards people with disabilities *t(*447) = 0.37, *p* = 0.71).

However, there was a statistical difference between the science majors (M = 3.06, SD = 0.65) and those who studied humanities (M = 3.27, SD = 0.67) regarding the level of experience on disability provided by the universities *t(*447) = 3.37, *p* = 0.01. As can be seen, those specialized in humanities had more university-based experiences with disabilities.

To determine if there were statistically significant differences between the mean averages of the responses on each dimension of the study instrument, and on the study instrument as a whole, based on Grade Point Average (GPA), the researchers used a one-way ANOVA.

As can be seen, there were no statistically significant differences for the level of awareness on disability (*p* = 0.07), for the level of awareness on the rights of people with disabilities (*p* = 0.54), the level of awareness about society's role towards people with disabilities (*p* = 0.20), and the level of experience provided by the universities (*p* = 0.71).

## 4 Discussion

The results of question one show that the relegation of items 1, 2, 3 (i.e. “distinguishing the various types of disability”, “awareness of disability groups” and “thinking that all persons with disabilities constitute one group”) to the 6, 8, 12 ranks, respectively, that there is a need to enhance awareness among Saudi graduates. This view is also supported by the transition of item 12 (“feeling the need to increase one's knowledge of disability categories”) to the fifth rank based on the analysis of the answers. According to Nor Azizi et al. (2018), there is a need to increase the awareness of disability among the public in general and university students to decrease any type of discrimination that is based on disability. In addition, Bunbury (2020) states that universities need to enhance the awareness of disability among their students and staff in an attempt to achieve an inclusive curriculum and environment. In fact, some universities may advocate the rights of students with disabilities on their campuses and tend to provide inclusivity to them, however, many universities do not consider awareness about disability as a priority and they need to provide some awareness programs in their curricula about disability and people with disabilities (Heymani et al., 2020).

The results of question two concentrated on awareness of the rights of people with disabilities. From these results, it is contended that there is a need to enhance awareness of the rights of people with disabilities. The frequency of the neutral option among the sampled respondents is due to their inadequate awareness of the qualitative as well as quantitative rights of these people. This view is supported by the responses of the sample respondents to the last two items 8 and 9 (“believing that people with disabilities can benefit from modern technology” and “believing that all people with disabilities depend on the help of others”), which go in the opposite direction of the scale, and which – in addition to the answers of the items 1 and 2 (i.e. “distinguishing the various types of disability” and “awareness of disability groups”) – reveal a reasonable level of awareness of the rights of the people with disabilities, yet also a need to increase awareness of those rights qualitatively and quantitatively. In this regard, Choi (2020) states that developing intervention programs in universities about the awareness of the rights of people with disabilities enhances positive attitudes towards people with disabilities.

In addition, the results of question three show that the level of awareness of the role of society towards people with disabilities is inadequate in terms of type and quantity. This result is substantiated by the fact that the general average of this axis settles on the neutral degree, and reflects a need to enhance the awareness of the role of community institutions. Besides, this interpretation is validated by the fact that the sample respondents responded with “strongly agree” to items 27 and 29 (“the importance for everyone in society to realize their responsibility towards people with disabilities” and “the importance to continue medical diagnosis and treatment for people with disabilities”): this reveals their awareness of the needs of people with disabilities and meanwhile their inadequate awareness of the role of community institutions toward people with disabilities and their rights (type, quantity and quality). Indeed, the UN encourages all governments to include awareness-raising programs in their education system about the role of social institutions and organizations in terms of enhancing human rights in general and the rights of people with disabilities especially (Fraser, 2020). These repeated and continuing demands hint that there is a shortage in the education systems, including the university level, about the awareness of the role of society towards people with disabilities.

Furthermore. the answers to question four show the general average of the items of this axis trending to a neutral degree. This can be attributable to the inadequate awareness among the respondents regarding the disability expertise the university can provide, both quantitatively and qualitatively. On the other hand, this interpretation can be perceived as evidence of inadequate providing of this expertise in Saudi universities. This view is supported by the fact that the respondents had the option not to agree on items 8 and 9 (“believing that people with disabilities can benefit from modern technology” and “believing that all people with disabilities depend on the help of others”): the former reflects the need for expertise in terms of type, and the second reflects the need in terms of quantity. Both revolve around the study plan and the number of courses in the syllabus. In this matter, many universities do not provide any type of awareness programs about disability and people with disabilities (Osman et al., 2015).

Finally, the results of question five show that there are differences in favor of public universities, which can be attributed to the following:

• Saudi public universities are greater in number and more widespread than private universities and are therefore more effective in terms of the number of students.
• Most Saudi public universities have faculties of education, humanities and social sciences, including special education, and thus offer study subjects closely related to disability.

In terms of gender, there were statistically significant differences among graduates of Saudi universities in favor of male respondents regarding awareness of the role of society towards people with disabilities. That can be attributed to the Saudi culture where Saudi males’ chances of exposure to and interaction with the community and institutions are far more than those of females. Males shoulder responsibility earlier than females. Besides, males have more prospects of mobility, job opportunities, and social relations than females do.

In terms of university major, statistically, significant differences were observed in favor of theoretical disciplines due to their scientific and objective relevance to special education, i.e., to which this study belongs. Besides, theoretical disciplines are greater in number and commoner among study disciplines/majors in Saudi universities.

Regarding Grade Point Average (GPA), there were no statistically significant differences between the answers of the sample respondents which can be attributed to this variable.

### 4.1 Strengths, limitations and implications of the study

The study has a number of strength sides. For instance, it is a scientific step to investigating the phenomenon of awareness about disability on a large scale in Saudi universities. Furthermore, this study may be used as a scientific reference for those interested and responsible for developing education plans and curriculum, especially concerning universities, to provide content that enhances awareness of disability among university students. In addition, this study was not limited to the apparent description of the level of awareness of disability but rather studied more in-depth aspects in the description and investigation of the level of awareness, including the rights of people with disabilities and the role of society.

However, this study has its limitations. First and foremost, it is limited to the Saudi context, and as such it may be expanded to include other countries or regions. Besides, as a result of limited time and resources, using the questionnaire as the primary method for gathering data. In other words, while technology can make it easy to collect data, face-to-face contact with respondents could have provided more insights. Another limitation is the investigation of only four variables; it is advised to consider more variables in future studies.

## 5 Conclusions

The study was conducted in Saudi Arabia with a sample of 449 Saudi university graduates to examine their level of awareness about disability and also the level of their experience with disability as provided by their universities. The findings suggest that there is an urgent need to enhance the level of awareness of disability among university students, including awareness of the rights of people with disabilities, and the role of society towards them. In addition, disability awareness programs are absent in Saudi universities and there is a need to include some educational programs for universities to enhance disability awareness. Moreover, the results reflect the desire of the study sample and consequently the study community to activate the scientific, educational and social programs offered by the university to increase their level of disability awareness.

On other hand, the results of the study showed that despite the need to increase the level of disability awareness among graduates of Saudi universities, there was also a statistically significant difference concerning this need between graduates of public universities and graduates of private universities.

Finally, the findings also show that Saudi graduates of humanities had greater levels of awareness about disability than those with scientific majors.

## Declarations

### Author contribution statement

Abdullah Madhesh: Analyzed and interpreted the data; Contributed reagents, materials, analysis tools or data; Wrote the paper.

### Funding statement

This research did not receive any specific grant from funding agencies in the public, commercial, or not-for-profit sectors.

### Data availability statement

The data that has been used is confidential.

### Declaration of interest's statement

The authors declare no conflict of interest.

### Additional information

Supplementary content related to this article has been published online at https://doi.org/10.1016/j.heliyon.2022.e11647.

## Appendix A Supplementary data

mmc1 mmc1
