# Supplementary material for: Awareness of disability among saudi university graduates
Source: Heliyon. 2022 Nov 16;8(11):e11647. doi: 10.1016/j.heliyon.2022.e11647 (PMC9674535; doi:10.1016/j.heliyon.2022.e11647)
Supplement: mmc1 [file mmc1.pdf]

Dear Prof. David González-Gómez, Education Section Editor,

I am writing to you regarding manuscript No. HELIYON-D-22-02138R2.

After receiving the latest email from Heliyon that informs us about authorship requirements in Heliyon that require making substantial contributions to the manuscript, I decided to remove my name from this paper because I do not meet these requirements and only supervised the general structure of this paper and few small issues. As a result, please remove my name from this paper and leave the main and only first author Dr. Abdullah Madhesh.

Prof, Abdullah Al Motawa

Education College, Shaqra University, Shaqra, Saudi Arabia

aalmotawa@su.edu.sa

I do agree with this action.

Dr. Abdullah Madhesh
Special Education Department, Shaqra University, Shaqra, Saudi Arabia
E-mail: mdhsh@su.edu.sa
